# The active feedback program: bringing medical students out of the shadows

**DOI:** 10.1080/10872981.2021.1939842

**Published:** 2021-06-11

**Authors:** Matthew A. Edwardson

**Affiliations:** Department of Neurology, Georgetown University, Washington, DC, USA

**Keywords:** Stroke, medical education, neurology, feedback, medical student, physicians

## Abstract

Despite many advances in medical education, medical students continue to mostly shadow on inpatient rotations like Neurology. They seldom receive face-to-face feedback or mentorship from attending physicians. This results from not training attending physicians how to integrate medical students into clinical activities in a way that does not detract from patient rounds. The ‘active feedback program’ is a framework for inpatient rotations that immerses medical students in clinical activities with the attending physician providing mentorship and feedback that emphasizes brevity. Expectations are laid out early. Students pick up 2–3 patients, performing daily oral reports and focused neurological exams with immediate feedback. Feedback includes items to not only correct the treatment plan, but also improve the student’s oral presentation and neurological exam skills. Students also receive formal individual feedback twice during the rotation that includes constructive criticism and specific task-oriented praise. The active feedback program awaits formal testing, but seems to result in medical students learning at an accelerated rate. Neurology residents also appear to benefit by learning from critiques of the medical students and taking on higher level responsibilities. Patient rounds move quickly, leaving time for the attending physician to keep up with other obligations. As academic Neurologists we have a duty to transfer our skills to the next generation of physicians. If proven in future studies, wide adoption of the active feedback program will allow us to finally move medical students out of the shadows and come closer to achieving this noble goal.

## Introduction

During my two-week Neurosurgery rotation in medical school I found myself turned into a glorified fly on the wall in a short white coat. I was largely ignored save for fetching compact discs from the Neurosurgeon’s office to play in the operating room. Rarely he asked me anatomy questions like, ‘which part of the cerebellum shares a name with another part of the body?’. I could not answer, ‘the cerebellar tonsil!’, because my mind was clouded by an unhealthy fear of authority figures. During an aneurysm coiling procedure a nurse saw how eager I was to get involved and sent me to draw blood from the IV. In the process my head brushed up against the divider separating the patient’s head from the surgical field. To this day I do not know if I broke the sterile field. My only feedback was a searing glance from the attending Neurosurgeon filled with disgust and contempt. Little did I know that my Neurosurgeon was battling 2 unseen enemies that likely prevented him from providing the mentorship and feedback I desperately needed during the rotation.

The first enemy was fierce competition for his time. Fast forward nearly 20 years later and now I am an attending Neurologist at a comprehensive stroke center. During inpatient weeks I juggle clinical care for 10–20 stroke patients, telestroke calls, keeping my research studies afloat, grant deadlines, helping care for 2 young children at home and sleep deprivation. You may face other pressures such as administrative and formal teaching responsibilities. As much as I would hope that clinical rotations for medical students have improved in the last 2 decades, if anything attending physicians today have even less time to provide clinical teaching and feedback. Indeed, the medical students I work with suggest the fly on the wall experience remains common. Given the time constraints that academic physicians currently face, we must adopt effective teaching strategies that stress time management.

The second enemy that my attending Neurosurgeon likely faced was lack of training in how to provide effective mentorship and feedback to students. Prior studies on feedback in education led to strategies that maximize the ability of feedback to impact behavioral change [1–21]. The Michaelson and Shultheiss model, for example, suggests that helpful feedback is specific, honest, timely and usable [1]. Feedback is most effective at the task level, followed by the process used to carry out the task, self-evaluation, and finally at the personal level [7]. Non-specific praise directed at the self, i.e., ‘you did a great job’, almost always inhibits the desired behavioral change [2,7,8,15,20]. This is not to say that all positive feedback is bad. Positive feedback that helps to foster self-efficacy specifically directed at the task enhances effort [2,7]. Students are also more satisfied with feedback if it includes at least some aspect of praise [6,7,15]. More recent models in medical education have tried to shift the dynamic so that the attending physician is not seen as an evaluator, but rather as a coach [19,21]. Receiving feedback from an evaluator takes on negative, stress-inducing connotations that relate back to the self, whereas a coach is focused on the task or process at hand and helping the learner achieve certain goals [21].

Many academic medical centers now offer courses on how to provide feedback to trainees [3,22]. What these courses often lack is guidance on how to efficiently integrate these strategies into an already packed schedule. The techniques described in this article couple some of the most helpful feedback strategies from the medical education literature with practical advice on how to deliver feedback efficiently during inpatient rotations. These techniques are integrated into an ‘active feedback program’ in which medical students are immersed in clinical activities early, receiving frequent feedback. Brevity is stressed at the beginning of the rotation, resulting in efficient patient rounds. The payoff for adopting the active feedback program is engaged medical students who seem to learn at an accelerated rate, fulfilling your calling as an educator with minimal impact on other time commitments.

## Active feedback program

### Layout expectations early

**Figure 1.**
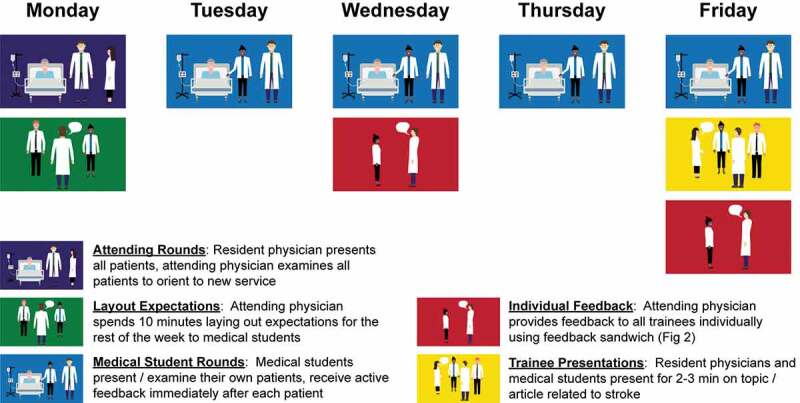
The active feedback program. this week-long program can be extended for longer rotations by keeping the 1^st^ individual feedback session halfway through the rotation. medical students at our institution may or may not round on the weekends, which is why this schedule ends on a friday

I was given no direction on expectations at the beginning of my Neurosurgery rotation and most other rotations in medical school. This left me floundering, trying to read the mind of the attending physician while gleaning tidbits from the residents and nurses. To prevent this situation with students on the inpatient Neurology rotation today and to ensure efficiency, layout expectations early. On day 1 at the end of rounds, sit down with the medical students and spend 5–10 minutes laying out expectations. Instruct each student to pick up 2–3 patients for whom they will provide a daily oral report and neurological exam. Tell them exactly how you like to hear daily patient presentations (Table 1), stressing points that foster brevity. For example, presentations are limited to 2–3 minutes and students should state only: major overnight events, abnormal exam findings (pertinent positives) while omitting normal exam findings, abnormal lab values, new diagnostic study results, and active problems during the assessment and plan. Tell students you will be coaching them during the rotation by providing brief feedback on their oral presentations immediately after delivery and more formal individual feedback twice during the rotation. The neurological exams are very focused, limited to only 2–3 neurological systems based on the area of brain injury. Finally, have them prepare a 2–3 minute talk for the last day of the rotation on a topic related to Neurology that also ties into their future career such as a landmark trial. Stress that these talks are informal, delivered in the middle (or end) of patient rounds, with no notes or handouts. By laying out expectations up front students have a solid idea of what the week will hold (summarized in Figure 1). In addition, they know the importance placed on brevity, which is the only way immersing students in clinical activities works with a busy schedule.

**Table 1.** Oral instruction to medical students on day 1 when laying out expectations on how to efficiently present stroke patients on inpatient rounds

Limit patient presentations to no more than 2–3 minutes
Short one-liner that includes patient age, sex, stroke type and location
Major overnight events
Blood pressure range over last 24 hours rounded to the nearest 10. Only include other vital signs if abnormal (febrile, tachypneic, etc.)
Exclude mention of the general medical exam unless abnormal (heart murmur detected or rales on chest auscultation, etc.)
Present the neurological exam in the order below, describing only pertinent positives (abnormal exam findings), while leaving out normal neurological systems and findings
-Mental status
-Speech
-Cranial nerves
-Motor
-Sensory
-Reflexes
-Coordination
Gait (which often cannot be tested depending on the severity of the stroke impairment)
Abnormal lab values and drug levels
New results from diagnostic tests
Problem-based assessment and plan as outlined below:
-Repeat one-liner, but this time include major stroke risk factors and other major active problems
-Discuss the stroke as the first major problem, being sure to talk through the suspected stroke etiology
-Discuss other major problems in turn. Do not discuss chronic problems that are not active or applicable to this hospitalization
-Disposition: describe any barriers to the patient transitioning to the next phase of recovery (stepping down from ICU-level care, discharging to home, acute rehabilitation, or nursing facility, etc.)

### Active feedback during patient rounds

For better or worse students are judged primarily on their ability to deliver succinct, well thought out oral patient presentations. Why then do they receive so little feedback on this critical skill? Most attending physicians correct the parts of the assessment and plan they disagree with, but there are many more dimensions to a good oral presentation. Was the student looking at their notes the whole time or did they have good eye contact? Was the presentation too long because the student repeated themselves or included too many unimportant details? Did they use the correct terminology to describe neurological exam findings and do so in a succinct manner? Do they boldly take ownership of the proposed assessment and plan or meekly offer up ‘suggestions’ for you to consider following if you ‘happen to agree’? Do they understand the suspected stroke etiology, or are they using nonspecific terms like ‘thromboembolic’, which most students use to describe the cause of all strokes? I argue that these other aspects of the oral presentation related to confidence, brevity, terminology, ownership and understanding are just as important to a good oral presentation as having the correct treatment plan. The next challenge is how to teach students to correct these deficiencies without disrupting patient rounds.

The best time to provide feedback on student oral presentations is immediately after delivery [7,8]. Immediate feedback tends to be more effective than delayed feedback [7], particularly for verbal, procedural and motor skill acquisition [8]. You are going to correct the parts of the treatment plan you disagree with anyway, so why not add a few more comments to foster their presentation skills while fresh in your memory? First let the student get through their entire patient presentation without interruption [23]. Ask the Neurology resident if they have anything to add. Finally provide your own feedback, correcting the treatment plan, but spending just as much time on comments to improve the student’s presentation skills. Describe no more than 2 items for them to correct and consider adding something specific that they did well. This strategy of immediate feedback goes against the adage, ‘praise in public and criticize in private’. In my experience, however, medical students do not mind being under the microscope in public as long as criticism is not heavy-handed. In fact, most comment on how much they learned about improving their own presentations by listening to critiques of fellow students.

The neurological exam is one of the most difficult skills for medical students to learn [24], yet few are given the opportunity to practice in front of an attending physician [10]. This again results from a lack of time and patience. Who wants to watch the medical student fumble through reflex testing for 10 minutes when the Neurology resident can demonstrate all pertinent exam findings in 2? The key is to keep the student focused on only 2–3 of the most important neurological systems and coach them through the exam in the interest of time. For stroke patients I typically have them perform the motor examination in combination with testing either speech, visual fields, or sensation/neglect depending on the area of brain injury and previously known deficits. Just like with their oral presentations, deliver on-the-spot feedback to correct any deficiencies and explain any subtleties related to the exam findings. In my experience, many students operate at the level of a junior Neurology resident after only a few days in the active feedback program.

### Formal individual feedback twice during the rotation

I did not perform to the best of my abilities during my Neurosurgery rotation in medical school, but on the last day I hoped to at least receive feedback on how to improve in the future. To my surprise, my attending Neurosurgeon was not even in the hospital that day. Most of the Neurosurgery attendings and senior residents were at a series of off-site lectures. How good of them to warn me. Ironically, this turned out to be a blessing in disguise. A visiting professor undergoing a yearlong fellowship was the only attending in the operating room that day. He let me scrub in, put in a Foley catheter and even assist him through the scalp incision and early parts of an aneurysm clipping procedure. This positive experience did little to change my final written evaluation. The wording is not in my official transcript, but I distinctly remember a tepid evaluation from my attending Neurosurgeon that included the word ‘unmotivated’. Experiences like mine where medical students receive no face-to-face feedback remain common today.

I consider formal individual feedback to medical students so important that I deliver it *twice* during the rotation. Why? There is nothing worse than hearing at the end of the rotation that you are deficient in some skill and then having no opportunity to correct it. Education research also supports the use of more frequent feedback to achieve the desired behavioral change [2,3,18,21]. The most efficient time to deliver individual feedback is immediately after patient rounds. Take each person out to the hall of the hospital ward or nearby waiting room where no one else is within earshot, starting with the senior resident and working your way down to the medical students by level of seniority. Instruct those waiting to receive individual feedback to stay on the hospital unit and start their post-rounds work until they are called out individually. If feedback is delayed until later in the day it will cut into your schedule as you try to round up residents and students strewn all over the medical campus. There is no getting around the fact that giving individual feedback to all trainees does take time out of your busy schedule. However, some of this lost time is regained by providing feedback on techniques to improve brevity, thereby increasing efficiency on rounds in future days.

### Content delivery during individual feedback sessions

Structure the individual feedback sessions in the following manner: student or resident self-evaluation, provide feedback to the trainee, ask them to evaluate you, and finally answer any remaining questions and provide encouragement. Begin by asking the trainee how they would rate their own performance on the rotation thus far including what they are doing well and what they could improve upon [3,23]. Self-evaluation serves 3 purposes: it helps you understand whether the trainee has insight into their own strengths and weaknesses, eases the mental burden required for you to provide feedback, and helps you see things from their perspective so you can tailor your feedback toward a common goal. I often have a difficult time coming up with specific items to correct or praise when the stroke service is busy and there are 4–5 rotating medical students. The trainee often mentions something that I did not think of, but is nonetheless an important skill that I can expand upon when delivering my own feedback. By tailoring your feedback to their self-evaluation they will be more invested, which is more likely to result in improved performance [19,21].

Your feedback to the trainee is the crux of the individual feedback session. Feedback is emotionally charged by nature, even more so to medical students and residents whose self-worth can be completely wrapped up in their performance [17]. Thus, it can helpful for feedback to include both constructive criticism and specific praise [1,4]. The feedback sandwich [5] consisting of praise, critique, praise has fallen out of favor [15,21,23]. Instead I commonly employ the ‘open-faced’ feedback sandwich where the corrective items are presented first [1,15]. Start with 1–2 items to improve upon. Limiting the number of items to correct keeps the trainee from getting overwhelmed and mitigates too many negative emotions. Almost all students can improve their oral presentation skills through less reliance on notes. My constructive feedback often includes a challenge to deliver oral reports by memory, rehearsing in the bathroom prior to rounds if necessary, and only using notes for lab values. Eliminating notes has the added bonus of improved efficiency during patient rounds. Conclude your feedback with the one aspect of their performance that was truly outstanding. Be sure that the praise is specific and directed at the task or procedural level and not at the person [1,2,7,8]. Saying, ‘You are a wonderful medical student’, will cause the trainee to focus on themselves and place less importance on fixing the deficiencies previously mentioned. On the other hand, ‘I was extremely impressed by the improvement in your exam skills throughout the rotation – you performed a coma exam on Mr. Smith today at the level of a Neurology resident’, helps reinforce their efforts for continued self-improvement. Ending the feedback sandwich with specific praise sends the trainee out charged with positive energy, motivated to correct the deficiencies brought to their attention.

Of course, feedback does not need to be so proscriptive. Some have suggested eliminating rules that balance negative and positive feedback altogether [15,21]. This is partly based on multiple studies suggesting that praise either has no effect or dampens the impact of corrective feedback [2,6–8,12,15]. It should be noted that in most of these studies the praise was directed at the personal level instead of the task level, which no one advocates. In the shift to more of a coaching paradigm in medical education, emphasis is placed on the relationship between the educator and the trainee [19,21]. By building trust through repeated encounters and working toward common goals they theorize that trainees are better equipped to receive corrective feedback [21]. In the course of a 1-week inpatient Neurology rotation this trust can be difficult to build. I therefore continue to include specific, task-oriented praise with most trainees during their individual feedback sessions.

Wrap up the individual feedback session by asking the trainee to evaluate you and by helping them navigate the next steps in their journey. Ask the trainee what they like about the rotation so far and what could be better. While trainees are often too timid to provide negative feedback to their attending physician, it can be illuminating. I was once told to learn the names of the nurses, which helped me better integrate the nursing staff into our daily rounds. End the mid-rotation feedback session by making sure the trainee has settled on an appropriate topic/article to present on the last day of the rotation. End the last feedback session by briefly discussing the trainee’s future career goals and providing further encouragement.

## Payoff

Deploying the active feedback program leads to a fully engaged medical team, achieved in a manner that does not detract from your other responsibilities. I frequently hear feedback from students like, ‘I learned more this week than any other rotation in medical school’, or ‘this is the first time I’ve ever performed or received feedback on the physical exam from an attending physician’. While this anecdotal praise from students and my opinion that that the active feedback program accelerates learning do not prove this is the best paradigm for clinical rotations, there can be no doubt that it is superior to shadowing. The active feedback program is also very efficient. We almost always finish rounds and all individual feedback/end of week presentations before noon (within 3 hours) unless interrupted by multiple stroke codes. This leaves at least some time each afternoon to keep up with my research and other non-clinical obligations. A neurologist who implemented a somewhat similar immersion of medical students in the outpatient setting found it led to high student ratings and only a negligible decrease in RVU production [10]. Although the active feedback program takes some attention away from the Neurology residents, their education does not suffer.

In fact, the active feedback program seems to benefit Neurology residents in many ways. Although they present fewer oral reports and less frequently examine patients in front of the attending physician, they often learn more from critiques of the medical students than they would otherwise. For example, I am less likely to correct a Neurology resident when it comes to the neurological exam unless they are plainly doing something wrong. This stems from thinking the resident possibly learned a different technique from another attending physician coupled with a small case of imposter syndrome [25] on my part. I have no qualms, however, about correcting a medical student and explaining in depth why I perform the exam a certain way. I am amazed at how many residents tell me they learned a great deal about improving their own neurological exam and did not understand the subtleties until I explained it to the medical students.

Neurology residents also benefit from the active feedback program because it effectively moves them up the chain of command. The junior Neurology resident is no longer burdened by having to perform an oral report and physical exam on all patients during rounds. This frees them up to take on some of the managerial and decision-making responsibilities of a senior resident. I try to treat them like senior residents. Allow the junior Neurology resident to provide feedback on each student’s treatment plan before you interject. When there are multiple ways to treat a patient, such as choosing between equally effective anti-hypertensive drugs, allow the junior resident’s opinion to stand. Of course, there is no getting around the fact that junior residents must write all of the patient notes and perform most of the legwork related to admitting and discharging patients. However, more fully integrated medical students become better liaisons to patients and their families, granting the junior Neurology residents more time to take on higher level responsibilities. Over the last 4 years my mean overall ratings from the Neurology residents were on par with the other Neurology faculty (n = 55, 4.79 vs. 4.74, where 4 is ‘excellent’ and 5 is ‘outstanding’), with higher ratings for effective use of literature (4.84 vs. 4.73) and providing constructive feedback (4.84 vs. 4.62).

Most aspects of the active feedback program, including the benefits, should also translate to surgical services. Consider how different my experience on the Neurosurgery rotation in medical school would have been had the attending Neurosurgeon laid out expectations early and employed some of these active feedback techniques. Simply substitute the immediate feedback after oral presentations described above with immediate feedback after performing tasks helpful in the operating room. A medical student won’t be clipping any aneurysms, but they can help with preparatory tasks. These might include tasks like those I performed on the last day of the rotation with the visiting professor: placing a Foley catheter, getting ancillary equipment in place and possibly assisting in the early stages of a craniotomy. By administering immediate feedback daily and one-on-one feedback mid-rotation that includes both constructive criticism and task-oriented praise, the medical student would quickly turn into an asset rather than a liability in the operating room. This might free up the surgical residents for less menial tasks. As with the suggestions on the Neurology service, feedback on surgical services must include items that improve efficiency.

## Conclusions

Medical students remain short-changed on clinical rotations due to lack of attention and mentoring from attending physicians. The attending physicians like the Neurosurgeon I encountered in medical school, however, are not entirely to blame. They juggle many other priorities and were simply never taught how to fully integrate medical students into clinical activities and provide feedback in a time-efficient manner. Effective feedback is an iterative process where you coach the trainee preferably toward a shared goal [3,7,8,10,18,21]. It must include instruction on how to correct deficiencies, but can also include specific task-oriented praise to increase motivation and effort [2,7,8]. The recent trend in medical education to shorten preclinical training while extending time in clinical rotations [26] will have minimal impact if students continue to merely shadow. Some students may receive no other formal training in Neurology and other clinical subspecialties through their entire medical career [27]. The active feedback program awaits formal evaluation to determine whether it truly enhances learning. If proven, we should widely adopt the active feedback program to improve the quality of the next generation of physicians. Adopting this program does not detract from our other obligations and fulfills our calling as clinician educators.
